# Robust Localization of Autonomous Excavators via EKF-Based Sensor Fusion in Real-World Construction Environments

**DOI:** 10.3390/s26206438

**Published:** 2026-10-12

**Authors:** Ahmet Yuce, Kenan Isik

**Affiliations:** Department of Mechatronics Engineering, Faculty of Engineering and Natural Sciences, Karabuk University, Karabuk 78050, Türkiye; ahmet@smartuniversal.com

**Keywords:** autonomous excavator, multi-sensor fusion, extended Kalman filter (EKF), robot localization, construction robotics, outdoor navigation

## Abstract

Reliable localization is a prerequisite for autonomous excavation, yet outdoor construction environments introduce GNSS degradation, vibration, and terrain-induced slip that can compromise single-sensor estimates. This study reports the implementation and field evaluation of an EKF-based localization system that fuses Global Navigation Satellite System–Real-Time Kinematics (GNSS-RTK), inertial measurement unit (IMU), and encoder measurements within a robot operating system (ROS) pipeline for an autonomous excavator. The fusion strategy weights complementary sensor measurements according to modeled uncertainty and down-weights GNSS updates classified as degraded. Experimental evaluation on a mapped outdoor route shows that the fused trajectory remains stable under non-ideal operating conditions, including signal obstructions and high-vibration segments. The EKF-fused trajectory achieved a path-deviation RMSE of 0.139 m and a P95 path deviation of 0.213 m relative to the independently surveyed reference path. These results support the feasibility of probabilistic multi-sensor fusion for autonomous excavation tasks in challenging field environments.

## 1. Introduction

The construction industry plays an important role in every aspect of daily life. From housing to transportation, and from industrial applications to energy production, the construction industry provides convenience by reshaping the face of the Earth. This convenience is achieved by overcoming the challenges posed by nature, as great forces are required to build strong structures. Although machinery is used extensively, the construction industry still heavily relies on human labor and intelligence in many aspects of its operations.

Excavators are among the most commonly used machines in the construction industry [1], where the power of the machine is controlled by a human operator. The precision of operations performed using manually controlled excavators is limited by the abilities and performance of the operators. Working conditions inside enclosed excavator cabins, along with long working hours, can negatively affect operator performance. In addition to cabin conditions, the overall work environment is also a critical factor for safety. Earthmoving operations in toxic or hazardous areas can adversely affect operators’ health. Furthermore, construction sites are highly dynamic environments where machinery and workers are constantly moving, materials are being placed, and the terrain is continuously changing. Adapting to such environments while maintaining efficiency and safety can be more effectively achieved using autonomous systems rather than relying solely on human operators. Considering these safety concerns, autonomous excavators present a promising alternative to manually operated systems. This study evaluates the field performance of an onboard localization system that integrates GNSS-RTK, IMU, and track-encoder measurements for a tracked excavator, with swing-angle-based transformation of upper-structure measurements into the undercarriage frame.

Autonomous excavators utilize a range of sensors, including LiDAR, cameras, and GPS, to perceive their surroundings and operate effectively in dynamic construction environments [2]. Using this data, excavators can better adapt to diverse and changing conditions where multiple machines and personnel are present. Autonomous systems can proactively prevent collisions, maintain safe operating distances, and comply with designated safety zones. Through data-driven decision making, not only can safety be improved, but task optimization can also be achieved. In multi-machine operations, autonomous excavators can coordinate with other equipment, reduce rework by optimizing task sequences, and ultimately improve project timelines.

For mobile systems, operational precision depends on accurate localization. Therefore, although autonomous systems can improve efficiency and safety, overall performance is directly influenced by localization accuracy in the construction environment [3].

In indoor environments with flat surfaces and known structures, localization is relatively easier because proprioceptive sensors are less affected by environmental disturbances. In contrast, construction sites present challenging conditions with uneven terrain and unstructured environments. In such cases, reliable localization requires multiple sources of feedback. This is because no single sensor can maintain accuracy under all conditions encountered in construction fields. Each sensor, however, has its strengths and weaknesses [4]. Common sensors used for outdoor localization include GNSS, IMUs, encoders, LiDAR, and cameras.

Among these, GNSS plays a critical role in outdoor localization. A GNSS determines position using satellite signals. Common systems include GPS, Galileo, GLONASS, and BeiDou. These systems estimate the receiver’s position by processing signals from multiple satellites. The fundamental principle of GNSS is three-dimensional positioning using signals from at least four satellites [5].

To further improve accuracy, real-time kinematics (RTK) is often employed. With RTK, centimeter-level accuracy can be achieved [6]. This technique enhances GNSS accuracy by incorporating correction data from a base station [7]. However, GNSS-RTK performance can be degraded by obstructions such as buildings, trees, and tunnels [8].

While GNSS provides precise positioning, orientation (roll, pitch, yaw) is also required for autonomous operation. For this purpose, inertial navigation systems (INSs), also known as inertial measurement units (IMUs), are commonly used [9]. IMUs combine accelerometers, gyroscopes, and magnetometers to measure motion-related quantities, such as acceleration, angular velocity, and orientation. These sensors enable real-time tracking of vehicle motion [10]. However, IMU-based orientation estimation can be affected by magnetic disturbances, which may introduce errors.

To mitigate such issues, alternative sensor fusion approaches are used. For example, GNSS-camera integration can improve robustness in environments where GNSS signals are unavailable, such as tunnels [11,12]. In such systems, RTK-enabled dual-antenna GNSS provides orientation, while vision-based methods compensate for GNSS signal loss [13].

IMU measurements are also subject to noise and drift, especially when integrating acceleration to estimate velocity and position [14]. To reduce these errors, encoders are often used alongside IMUs. Encoders convert wheel rotations into velocity estimates, enabling improved estimation of linear and angular motion [15]. However, encoder accuracy depends heavily on ground conditions, and issues such as wheel slip or loss of contact can lead to unreliable measurements.

The data from the sensors mentioned above must be processed to achieve the most accurate outputs. Sensor fusion algorithms can optimize the positioning process of autonomous vehicles by combining data from different types of sensors. These algorithms typically use filtering techniques (e.g., Kalman filtering) or artificial intelligence-based methods [16] to integrate sensor outputs and produce more accurate results.

In environments with static obstacles, different localization methods can be applied depending on the scenario [17]. When the environment contains fixed landmarks, Simultaneous Localization and Mapping (SLAM) is commonly used [18]. SLAM estimates position while simultaneously building a map of the environment, often using probabilistic methods such as particle filters [19].

The choice of sensors depends on the localization method. SLAM typically relies on LiDAR and cameras. However, its performance can degrade in highly dynamic environments. For instance, a robot operating in a crowded shopping mall may struggle due to constantly changing surroundings. Despite this limitation, SLAM remains widely used in outdoor and construction applications [20].

Another widely used method is the extended Kalman filter (EKF) [21], a Bayesian approach for estimating the state of nonlinear systems based on noisy and uncertain sensor measurements [22]. EKF combines measurements from multiple sensors to improve state estimation accuracy. The “extended” aspect refers to the linearization of nonlinear models, making it suitable for real-world robotic systems [23,24,25].

Established approaches already combine complementary sensors and adapt their use to changing conditions. Li et al. combine LiDAR-scan matching with GNSS, inertial data, and wheel encoders for urban delivery-robot localization, including a fallback strategy when the prior map becomes inconsistent [26]. Kaczmarek et al. use a planar GNSS/IMU/odometry EKF with measurement weights selected by RTK solution status, tested under open sky, partial obstruction, and near walls [25]. Che et al. integrate GNSS, IMU, odometer, and barometer measurements with sensor-availability switching and inertial-error estimation in urban vehicle trials [27].

Construction machinery presents additional challenges because GNSS visibility, terrain, and sensor quality vary across unstructured worksites. The HEAP walking excavator combines GNSS-RTK with inertial and kinematic measurements for real-world autonomous tasks [28]. Nubert et al. further fuse GNSS, IMU, and LiDAR in a dual-graph estimator that maintains consistent localization during GNSS loss and recovery, validated on two walking excavators [3]. This approach improves robustness but requires LiDAR and greater computational complexity than conventional filters.

Infrastructure-based alternatives can reduce dependence on GNSS. Inagawa et al. match a vehicle model to point clouds from worksite-mounted LiDAR sensors, achieving position and yaw accuracies of 0.121 m and 0.016 rad on rough terrain [29]. However, fixed external sensors limit deployment across changing or unmapped sites. Recent reviews therefore distinguish infrastructure-dependent, infrastructure-free, and hybrid methods while identifying dynamic, unstructured environments as a continuing challenge [30].

For tracked machinery, Reitbauer and Schmied combine dual-antenna GNSS, IMU, and encoders in a slip-aware error-state filter, evaluated on a compost turner against total-station trajectories with post-processed GNSS outages [31]. Although encoder data can bridge short degradations, excavators additionally experience track slip, uneven terrain, vibration, direction changes, and GNSS obstruction. These differences motivate dedicated evaluation of a simpler GNSS-RTK/IMU/encoder architecture on an operational excavator.

The framework employs established differential-track motion prediction and GNSS/IMU measurement updates, with heuristic covariance scaling according to GNSS quality and outage duration to adapt the relative contribution of the measurements under degraded GNSS conditions. The engineering contribution lies in integrating these established estimation methods with a URDF/ROS TF-based transformation framework that converts GNSS and IMU measurements obtained from the upper structure of the excavator into the undercarriage base_link coordinate frame using the measured swing angle. This integration enables globally referenced localization to be implemented on an operational excavator using onboard sensors without requiring LiDAR maps or external localization infrastructure. The experimental contribution is a field evaluation of the complete localization system against a surveyed reference path, with encoder-only dead reckoning as a baseline for the complete fusion system. On the evaluated route, the system achieved a path-deviation RMSE of approximately 14 cm and a P95 path deviation of approximately 21 cm relative to the surveyed reference path.

## 2. Methodology

In this section, we present the hardware we use for the implementation of our localization study and the methods we employ for achieving precise localization of the autonomous excavator we use in this project.

### 2.1. Hardware Setup

#### 2.1.1. The Excavator

**Figure 1:**
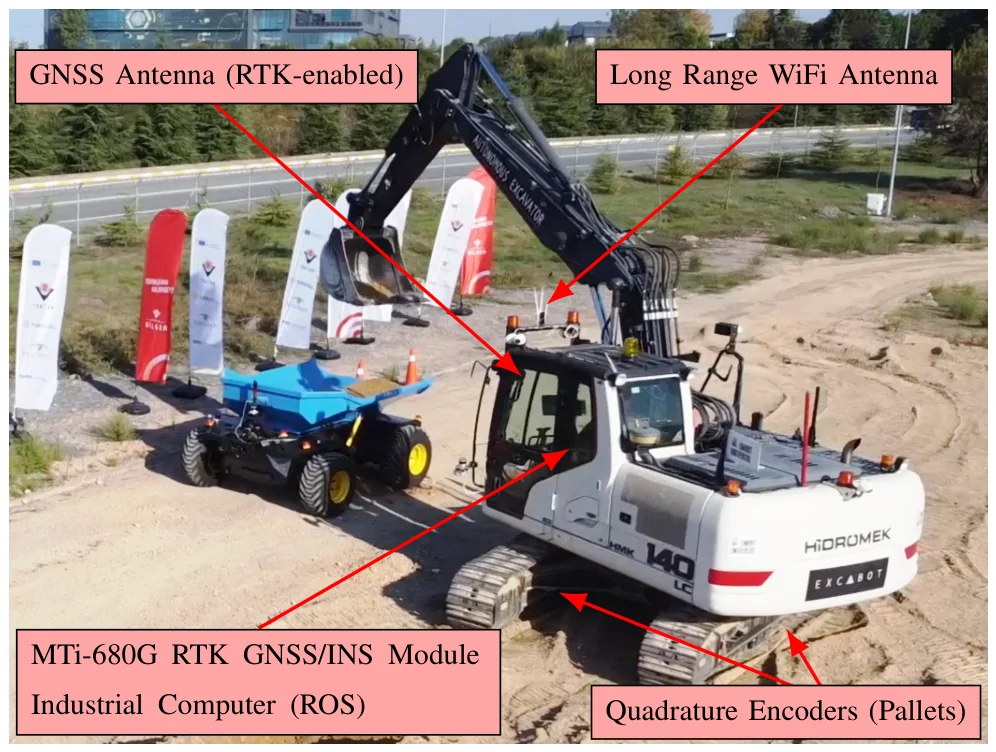
The HKM 140LC excavator platform used in this study, along with the integrated hardware components for autonomous operation. The figure highlights the placement of key elements, including the GNSS antenna mounted on the upper structure, the IMU unit installed inside the cabin, track encoders attached to the drive system, and the industrial onboard computer located inside the cabin.

The experimental platform used in this study is a 14-ton HKM 140LC hydraulic excavator manufactured by Hidromek (Ankara, Türkiye), a model widely employed in medium-scale excavation and construction operations, including projects within the European Union. The excavator provides a suitable testbed for autonomous operation due to its robust hydraulic actuation system and compatibility with external control architectures. Figure 1 illustrates the excavator platform along with the integrated hardware components used in this study.

To enable autonomous functionality, the excavator has been augmented with additional hardware and control layers. A microcontroller unit (MCU) is responsible for real-time control of the excavator’s hydraulic system. The MCU interfaces directly with the electro-hydraulic valves that govern both the digging mechanisms (boom, arm, and bucket) and the vehicle mobility (tracks). It executes control commands received from the high-level system and ensures deterministic actuation with appropriate timing constraints. An industrial-grade onboard computer located within the excavator cabin performs autonomous decision making and system coordination. This unit hosts the full software stack required for autonomy, including sensor data acquisition, preprocessing, state estimation, and control algorithms. In particular, the EKF-based sensor fusion algorithm is executed on this platform to estimate the excavator’s position and orientation in real time.

#### 2.1.2. Sensor Suite and Data Acquisition

In this study, a multi-sensor configuration consisting of both proprioceptive and exteroceptive sensors was employed to achieve robust localization of the autonomous excavator. Specifically, two proprioceptive sensors (encoders and an IMU) and one exteroceptive sensor (a GNSS-RTK system) were utilized to provide complementary measurements for state estimation.

##### Encoder-Based Odometry

For odometry estimation, two ATEK DS ARK 001 ARK S 58 rotary encoders (Kocaeli, Türkiye) (IP67; resolution: 1600) were installed, one on each excavator track. The track width is 1.99 m. Under ideal surface conditions, the excavator’s position and orientation can be estimated using differential kinematics derived from encoder measurements. However, in real-world construction environments, the reliability of encoder data is affected by factors such as uneven terrain, track slippage, and intermittent loss of ground contact. These effects can introduce significant errors in odometry, necessitating the use of additional sensing modalities for accurate localization.

##### IMU and GNSS-RTK Sensor Module

An Xsens MTi-680G RTK GNSS/INS module (Enschede, The Netherlands) was installed inside the excavator cabin, with the GNSS antenna mounted on the cabin roof. The module provides GNSS position and IMU motion data, which are combined with track-encoder measurements by the custom EKF to estimate the excavator pose.

A swing-angle sensor measures the relative yaw between the upper structure and the undercarriage. Its joint state updates the ROS URDF/TF transform from the upper-structure swing frame to the undercarriage base_link frame, allowing the cabin-mounted IMU and roof-mounted GNSS antenna measurements to be expressed relative to base_link.

##### IMU Data Characteristics

The IMU data are provided in a pre-fused format by the MTi-680G module, combining measurements from the gyroscope, accelerometer, and magnetometer. The resulting outputs include the following:

- **Orientation:** roll, pitch, yaw (in degrees or radians);
- **Angular velocity:** X, Y, Z axes (in rad/s);
- **Linear acceleration:** X, Y, Z axes (in m/s^2^).

These measurements enable real-time tracking of the excavator’s motion and orientation, although they remain susceptible to noise and drift over time.

##### GNSS-RTK Data Characteristics

RTK correction enables centimeter-level GNSS positioning under favorable open-sky conditions with a stable fixed solution. However, this performance is not constant for every measurement and can degrade with changing satellite visibility, correction availability, and multipath. Therefore, the EKF uses empirically determined measurement covariance and solution-dependent weighting, while Section 3 reports the resulting end-to-end path-deviation performance under the experimental conditions.

The GNSS data include the following:

- **Latitude, longitude, altitude:** Provided in decimal degrees and meters;
- **Position quality:** Centimeter-level under favorable RTK-fixed conditions and solution-dependent during field operation.

The GNSS antenna is mounted on the roof of the excavator cabin to ensure a clear line of sight to satellites, minimizing signal degradation caused by structural or environmental obstructions.

##### Communication and Data Transmission

To enable remote monitoring and control, a Wi-Fi antenna was installed on the excavator. This communication interface allows real-time data transmission between the onboard system and a remote control station, facilitating both system supervision and data logging.

The hardware components and their specifications are summarized in Table 1.

### 2.2. Sensor Calibration

Accurate sensor calibration is essential for reliable excavator localization, as each sensing modality is affected by distinct error sources in outdoor construction environments. Consistent with prior studies on multi-sensor outdoor navigation [4,7,8], calibration was performed at both the sensor and system levels before conducting EKF-based fusion experiments.

#### 2.2.1. GNSS-RTK Calibration

GNSS-RTK calibration focused on mitigating multipath effects and minimizing correction latency. The RTK base station was deployed in an open-sky location, and correction-link stability was verified before each run. The rover antenna, mounted on the excavator cabin roof, was positioned to reduce structural masking and signal reflections. Correction update parameters were tuned to maintain real-time operation while preserving high positional accuracy under sufficient satellite visibility [6,7].

#### 2.2.2. IMU Calibration

IMU calibration combined manufacturer-provided compensation with field verification. Static intervals were used to estimate residual accelerometer and gyroscope biases, while controlled maneuvers were performed to verify axis alignment and scale consistency. Magnetometer behavior was also evaluated for local disturbances. These steps reduced orientation drift and improved robustness during temporary GNSS degradation [9,10].

#### 2.2.3. Encoder Preprocessing

Quadrature encoder counts were unwrapped and averaged over a 10-sample window. Track speed was calculated using Equation (1) and low-pass-filtered using Equation (2). No additional encoder calibration was performed:

(1) $$v=\frac{\Delta e}{N}\frac{2\pi r}{\Delta t}$$

where Δe is the encoder count change over the elapsed interval Δt, *N* is the number of counts per revolution, and *r* is the effective rolling radius.

(2) $$v_{\mathrm{filt}}\left(k\right)=\alpha v\left(k\right)+\left(1-\alpha \right)v_{\mathrm{filt}}\left(k-1\right)$$

where α∈[0,1] is an online-tunable filter weight. Speed values outside ±0.7 m/s are rejected as outliers.

#### 2.2.4. Integrated Sensor Calibration

After individual calibrations, all sensor streams were synchronized to a common time base and expressed within a consistent reference frame. Temporal alignment reduced inter-sensor latency effects, while spatial alignment ensured consistent interpretation of position and heading measurements during the EKF update step.

Overall, these calibration procedures significantly improved the consistency and reliability of sensor measurements, directly contributing to enhanced localization accuracy under challenging operating conditions.

### 2.3. Preliminary Tests and Data Collection

Preliminary experiments were conducted to validate the calibrated sensor stack and to establish a repeatable field-testing protocol. Initial short runs were performed on soil surfaces with varying compaction levels to verify signal continuity, timestamp consistency, and basic motion consistency between the GNSS, IMU, and encoder data streams. The field runs were then extended to more challenging conditions, including uneven terrain, vibration, and periods of reduced GNSS quality.

An ROS-based logging pipeline was used to record GNSS-RTK, IMU, and encoder data, along with system timestamps and run annotations. In addition to localization signals, environmental context (e.g., ground conditions and weather notes) was documented to support the interpretation of performance degradation mechanisms.

The dataset used for quantitative evaluation corresponds to a mapped loop route consisting of straight segments, moderate-curvature paths, and sharp turns, with a total duration of approximately 363 s. This dataset includes non-ideal operating conditions and serves as a representative benchmark for evaluating the robustness and accuracy of the proposed EKF-based localization framework.

Data collected during the system-development tests were used exclusively for system calibration and parameter tuning. These parameters were fixed before the evaluation phase and remained unchanged thereafter. A separate dataset, not used for calibration or tuning, was then collected for quantitative evaluation.

### 2.4. Implementation of EKF

The EKF is a recursive estimator for nonlinear systems and is well suited for autonomous excavator localization, where both motion and sensing processes are nonlinear. The EKF approximates nonlinear models through first-order Taylor linearization around the current state estimate.

#### 2.4.1. State and Measurement Models

The excavator state at time step *k* is defined as

(3) $$x_{k}={\left[x_{k},y_{k},\theta_{k}\right]}^{T}$$

where $\left(x_{k},y_{k}\right)$ represent the excavator’s position in the global reference frame, and $\theta_{k}$ denotes its heading angle.

More precisely, $\left(x_{k},y_{k}\right)$ denotes the midpoint between the left and right tracks projected onto the ground (the undercarriage base_link origin), and $\theta_{k}$ is the undercarriage heading. The local map frame is right-handed, with *x* pointing east, *y* pointing north, and positive yaw measured counterclockwise from the positive *x*-axis. Although the GNSS antenna and IMU are mounted on the upper structure, their measurements are transformed into the undercarriage base_link frame through the ROS URDF/TF chain. The swing sensor supplies the relative upper-structure angle at each timestamp, so the sensor-to-base_link transformation is updated as the upper structure rotates.

The ROS body frame is car_body, used as base_link. The frame tree is map → odom → car_body → swing → boom → arm → bucket. The measured swing-joint angle updates the transform between the upper structure and base_link; the GNSS and IMU sensor frames are resolved through this transform before localization updates. The resulting base_link pose is represented in the local map frame registered to the surveyed site datum.

The calibrated GNSS antenna offset components are −0.90, −0.30, and +2.0 m, and the IMU mounting-yaw offset is 0.03 rad. These static mounting offsets are composed with the measured time-varying swing-to-base_link transform in ROS TF. The resulting body-frame lever arm $\ell_{G,k}$ and IMU yaw offset $\beta_{I,k}$ are updated for each measurement timestamp and are used in Equation (6).

The state transition model is given by

(4) $$x_{k}=f\left(x_{k-1},u_{k}\right)+w_{k}$$

where $w_{k}∼N\left(0,Q\right)$ represents process noise and $u_{k}={\left[\Delta s_{k},\Delta \theta_{k}\right]}^{T}$ is the odometry input.

Using differential-track odometry, the nonlinear motion model is expressed as

(5) $$\left[\begin{array}{c} x_{k}\\ y_{k}\\ \theta_{k} \end{array}\right]=\left[\begin{array}{c} x_{k-1}+\Delta s_{k}\cos\left(\theta_{k-1}+\Delta \theta_{k}/2\right)\\ y_{k-1}+\Delta s_{k}\sin\left(\theta_{k-1}+\Delta \theta_{k}/2\right)\\ \theta_{k-1}+\Delta \theta_{k} \end{array}\right]+w_{k}$$

where $\Delta s_{k}=\left(\Delta s_{r}+\Delta s_{l}\right)/2$ is the average displacement of the right and left tracks, $\Delta \theta_{k}=\left(\Delta s_{r}-\Delta s_{l}\right)/d$ is the yaw increment, and *d* is the track width. Forward displacement is positive for both tracks; $\Delta s_{r}$ and $\Delta s_{l}$ denote the right- and left-track displacements, respectively, so $\Delta s_{r}>\Delta s_{l}$ produces positive (counterclockwise) yaw.

The GNSS-RTK position and IMU yaw are processed as separate asynchronous measurements:

(6) $$z_{k}^{G}=h_{G}\left(x_{k}\right)+v_{k}^{G}=\left[\begin{array}{c} x_{k}\\ y_{k} \end{array}\right]+R\left(\theta_{k}\right)\ell_{G,k}+v_{k}^{G},\;z_{k}^{I}=h_{I}\left(x_{k}\right)+v_{k}^{I}=\theta_{k}+\beta_{I,k}+v_{k}^{I}$$

Here, $\ell_{G,k}={\left[\ell_{Gx,k},\ell_{Gy,k}\right]}^{T}$ is the effective horizontal lever arm from base_link to the GNSS antenna after composing the fixed sensor-to-swing mounting transform with the time-varying swing-to-base_link transform. The term $\beta_{I,k}$ is the corresponding effective IMU yaw offset. Both are obtained from ROS TF using the swing-angle measurement at time *k*. The matrix R(θ)=[cosθ−sinθ;sinθcosθ] rotates the lever arm into the map frame. The terms $v_{k}^{G}∼N\left(0,R_{G}\right)$ and $v_{k}^{I}∼N\left(0,R_{I}\right)$ represent GNSS-position and IMU-heading noise, respectively.

Latitude and longitude are converted to the local map using the test-site origin $\left(\phi_{0},\lambda_{0}\right)=\left(40.789575{}^{\circ},\;29.458488{}^{\circ}\right)$ and the fixed-scale approximation

(7) $$x=\left(\lambda -\lambda_{0}\right)\;84,281.9231,\;y=\left(\phi -\phi_{0}\right)\;111,132.92$$

where angular differences are in degrees and the resulting coordinates are in meters.

Equation (7) uses constant degree-to-meter scales at the test-site origin. The longitude scale is calculated as 111,320$\cos\phi_{0}$, while the latitude scale is 111,132.92 m/degree. Compared with the corresponding WGS-84 local scales, these values differ by −0.1425% and +0.0748%, respectively.

The surveyed path covers approximately 72.01 m × 59.51 m. Over this area, comparison with a WGS-84 ECEF-to-ENU conversion gives a maximum horizontal difference of 0.0858 m. Therefore, a standard ENU or projected-coordinate transformation is preferable when centimeter-level geodetic consistency is required.

#### 2.4.2. Linearization via Jacobian Matrices

The EKF linearizes the motion model using the Jacobian matrix

(8) $$F_{k}=\frac{\partial f}{\partial x}{|}_{x_{k-1}}=\left[\begin{array}{ccc} 1 & 0 & -\Delta s_{k}\sin\left(\theta_{k-1}+\Delta \theta_{k}/2\right)\\ 0 & 1 & \Delta s_{k}\cos\left(\theta_{k-1}+\Delta \theta_{k}/2\right)\\ 0 & 0 & 1 \end{array}\right]$$

The corresponding measurement Jacobians are

(9) $$H_{G}=\left[\begin{array}{ccc} 1 & 0 & -\ell_{Gx,k}\sin\theta_{k}-\ell_{Gy,k}\cos\theta_{k}\\ 0 & 1 & \ell_{Gx,k}\cos\theta_{k}-\ell_{Gy,k}\sin\theta_{k} \end{array}\right],\;H_{I}=\left[\begin{array}{ccc} 0 & 0 & 1 \end{array}\right]$$

#### 2.4.3. Noise Covariance Determination

The nominal covariances were selected empirically to balance prediction and measurement updates using data from the development tests, separately from the evaluation dataset, and the tuning parameters were fixed before evaluation. These values represent modeled uncertainties rather than independently validated sensor-noise statistics.

##### Process Noise (Q)

To characterize process noise, controlled slippage tests were performed on dry soil, wet soil, and mud. Encoder-based predictions were compared against RTK-GNSS reference trajectories under good satellite visibility. The resulting covariance matrix was defined as

(10) $$Q=\left[\begin{array}{ccc} 0.04 & 0 & 0\\ 0 & 0.04 & 0\\ 0 & 0 & 0.01 \end{array}\right]$$

The diagonal entries are variances of the additive prediction errors in *x*, *y*, and θ, with units m^2^, m^2^, and rad^2^, respectively. Their nominal standard deviations are 0.20 m, 0.20 m, and 0.10 rad (approximately 5.73°). The diagonal form assumes uncorrelated process-noise components. Hereafter, $Q_{\mathrm{nominal}}\equiv Q$. This covariance is defined for the nominal prediction interval $\Delta t_{\mathrm{nom}}=0.02$ s and is scaled at step *k* by $\Delta t_{k}/\Delta t_{\mathrm{nom}}$. The time-step ratio is dimensionless, so this scaling preserves the variance units.

##### Measurement Noise ($R_{G}$ and $R_{I}$)

The nominal measurement covariance values used in the EKF were selected empirically as

(11) $$R_{G}=\left[\begin{array}{cc} 0.0004 & 0\\ 0 & 0.0004 \end{array}\right],\;R_{I}=0.0009$$

The diagonal entries of $R_{G}$ are GNSS *x*- and *y*-position measurement-error variances, while $R_{I}$ is the IMU yaw measurement-error variance. These tuning values correspond to assumed standard deviations of 2 cm per horizontal axis and 0.03 rad (approximately 1.7°) in yaw. Hereafter, $R_{G,\mathrm{nominal}}\equiv R_{G}$. Its entries have units of m^2^, whereas $R_{I}$ has units of rad^2^.

#### 2.4.4. Dynamic Sensor Weighting Strategy

To handle GNSS degradation and outages, the covariance matrices are adaptively tuned.

(12) $$R_{G,\mathrm{adaptive}}=\left\{\begin{array}{cc} R_{G,\mathrm{nominal}}, & \mathrm{for}\;\mathrm{an}\;\mathrm{RTK}\text{-}\mathrm{fixed}\;\mathrm{solution}\\ 10\cdot R_{G,\mathrm{nominal}}, & \mathrm{for}\;\mathrm{an}\;\mathrm{RTK}\text{-}\mathrm{float}\;\mathrm{or}\;\mathrm{degraded}\;\mathrm{valid}\;\mathrm{solution}\\ \mathrm{GNSS}\;\mathrm{update}\;\mathrm{skipped}, & \mathrm{for}\;\mathrm{an}\;\mathrm{invalid}\;\mathrm{or}\;\mathrm{unavailable}\;\mathrm{solution} \end{array}\right.$$

The categories are obtained from the receiver-reported GNSS solution status. The IMU covariance $R_{I}$ is not scaled by GNSS quality, and IMU heading updates continue during GNSS outages.

During GNSS outages, process uncertainty increases; therefore, the process noise is scaled as

(13) $$Q_{\mathrm{adaptive},k}=\frac{\Delta t_{k}}{\Delta t_{\mathrm{nom}}}\left\{\begin{array}{cc} Q_{\mathrm{nominal}}, & \mathrm{if}\;\mathrm{GNSS}\;\mathrm{available}\\ 2\cdot Q_{\mathrm{nominal}}, & \mathrm{if}\;\mathrm{outage}<30\;s\\ 5\cdot Q_{\mathrm{nominal}}, & \mathrm{if}\;\mathrm{outage}\geq 30\;s \end{array}\right.$$

The covariance-scaling rules adjust prediction and measurement weighting according to GNSS quality and availability within the standard EKF architecture. The dimensionless factors 10, 2, and 5 multiply variances rather than standard deviations. They down-weight degraded GNSS measurements or increase predicted uncertainty during outages. The 30 s threshold determines which process-uncertainty inflation factor is applied.

The threshold was selected during development as an engineering breakpoint between short GNSS interruptions and prolonged encoder/IMU-only propagation. The controlled offline sweep in Section 3.7 evaluates outage durations of 30, 40, 50, and 60 s while holding this threshold and all other filter settings fixed. Thus, the sweep evaluates the effect of outage duration rather than the sensitivity to alternative threshold values.

The covariance multipliers and the 30 s threshold are engineering heuristics rather than physical limits or demonstrated optimal settings. Future work will assess their sensitivity and applicability to other operating conditions through controlled replays with identical inputs and one parameter varied at a time.

A GNSS update is available when the receiver reports a finite RTK-fixed, RTK-float, or other degraded-but-valid position. Invalid, unavailable, or non-finite positions are skipped. The outage timer measures elapsed time since the last accepted valid GNSS update, resets to zero after each accepted update, and changes the process-noise scale at 30 s.

The EKF algorithm, given in Algorithm 1, was implemented in ROS using the sensor topics /gps/fix, /imu/data, and /odom. The update rates are 4 Hz for GNSS, 30 Hz for the IMU, 75 Hz for the encoders, and 30 Hz for the EKF. Effective rates measured from the recorded streams are given in Section 3. The fused state estimate was published on the /odometry/filtered topic.   
**Algorithm 1:** EKF with adaptive noise covariance
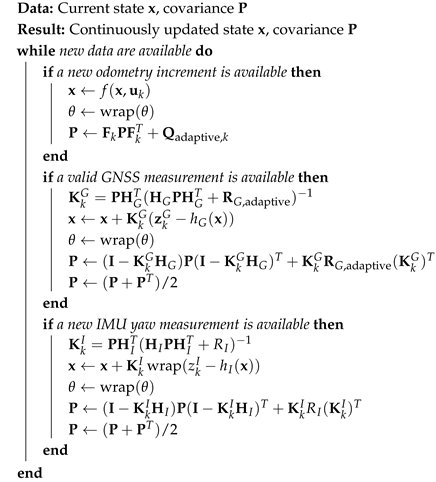


The filter is initialized from the first valid, time-aligned GNSS and IMU samples as $\theta_{0}=\mathrm{wrap}\left(z_{0}^{I}-\beta_{I,0}\right)$ and ${\left[x_{0},y_{0}\right]}^{T}=z_{0}^{G}-R\left(\theta_{0}\right)\ell_{G,0}$, with $P_{0}=\mathrm{diag}\left(0.0004,0.0004,0.0009\right)$. Sensor events are processed in timestamp order. Each odometry event triggers prediction using its measured $\Delta t_{k}$; GNSS and IMU measurements then update the most recent predicted state. The function wrap(α) maps angles to [−π,π).

**Figure 2:**
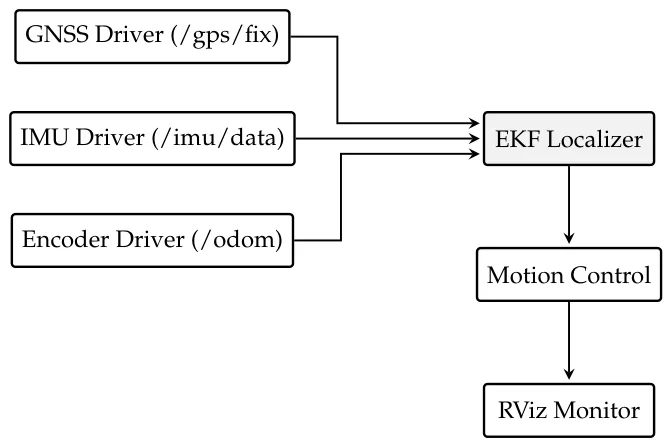
ROS-based localization architecture used in the EKF implementation. GNSS, IMU, and encoder drivers publish asynchronous measurements, which are fused by the EKF localizer to estimate the excavator pose. The estimated state is then utilized by the motion control module and visualized in RViz.

The overall ROS-based localization architecture used in the implementation is shown in Figure 2. The GNSS, IMU, and encoder measurements are processed asynchronously and fused by the EKF localizer to estimate the excavator pose, which is then forwarded to the motion control and monitoring modules.

**Figure 3:**
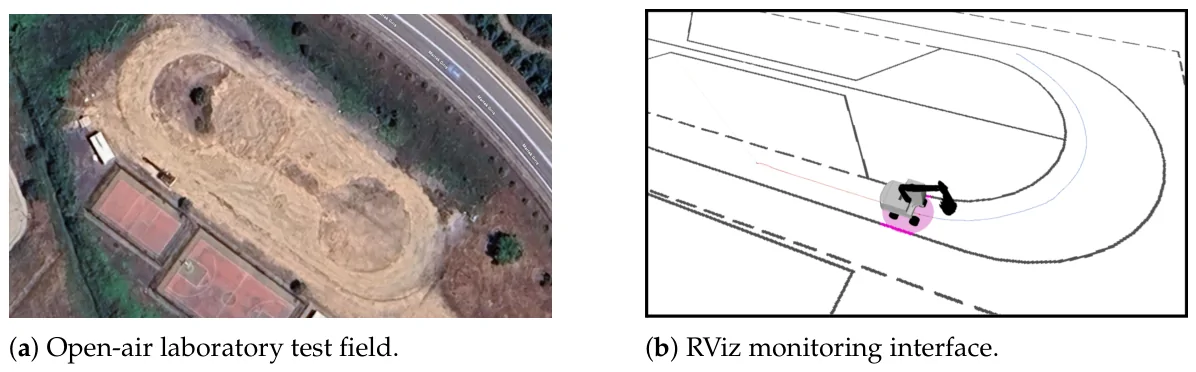
Experimental environment and online monitoring of the localization system. (**a**) The open-air laboratory field used for data collection. (**b**) The RViz display, which overlays the surveyed reference path, fused EKF trajectory, and raw odometry to qualitatively assess consistency, drift behavior, and correction performance during GNSS degradation periods.

Figure 3 summarizes the experimental context and online monitoring interface. The open-air laboratory test field used for data collection is shown together with the RViz interface employed as an online sanity-check tool during experiments. A stable overlap between the fused trajectory and the surveyed reference path indicates consistent filter tuning, while divergence between raw odometry and the EKF trajectory highlights the correction contribution of GNSS and IMU updates.

## 3. Results

### 3.1. Experimental Scenario and Evaluation Protocol

**Figure 4:**
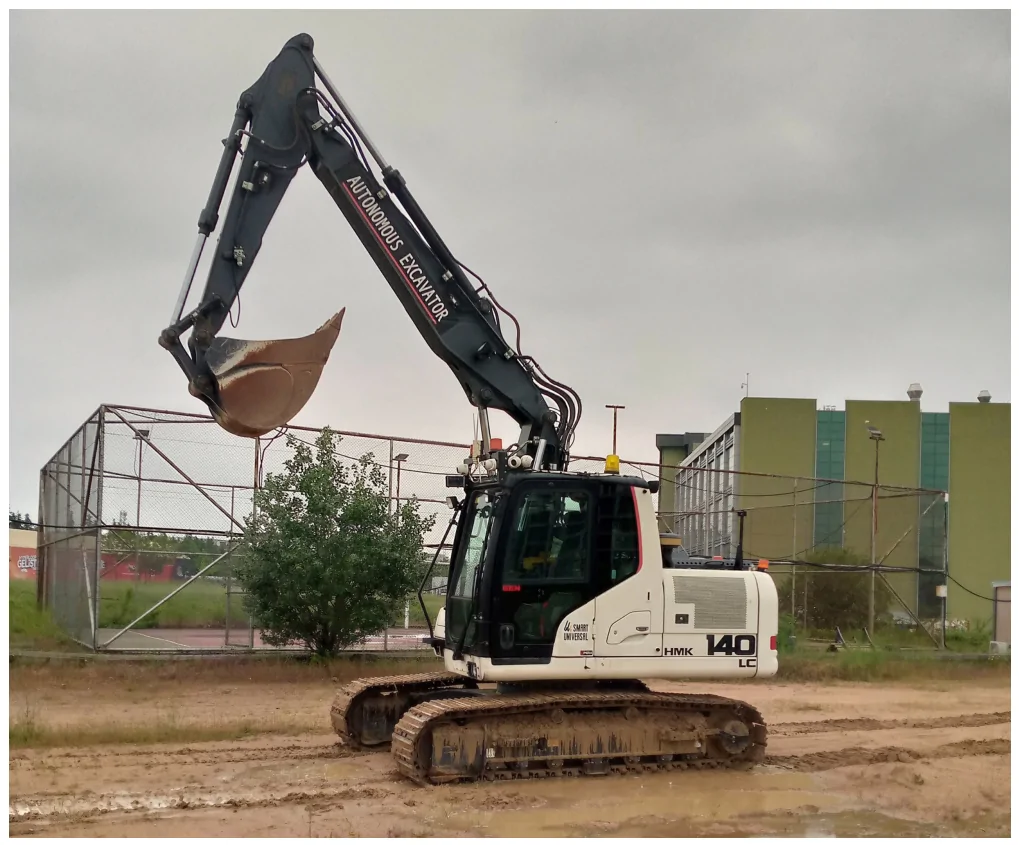
The HKM 140LC excavator platform in open-air laboratory test field on the test day. Testing was conducted in rainy weather on muddy ground.

The proposed localization framework was evaluated at a mapped outdoor field representative of a construction site. The loop-shaped route contained straight segments, moderate-curvature paths, and sharp turns. With assistance from geomatics engineering specialists, a local site datum was established using survey-grade GNSS equipment. Control points along the intended route were surveyed relative to this datum and transformed into the local Cartesian map frame. The piecewise-linear path connecting these points is herein termed the surveyed reference path and was established independently of the onboard GNSS-RTK, IMU, and encoder measurements used by the EKF. The recorded dataset spans approximately 363 s and includes non-ideal operating conditions such as surface irregularities, mechanical vibrations, and variations in GNSS signal quality. Estimated positions were evaluated by their shortest distance to the surveyed reference path. Because the reference is a static path rather than a time-synchronized sequence of true vehicle poses, the reported metrics quantify spatial deviation from the surveyed reference path, not time-synchronized absolute localization error. Figure 4 shows the system in the open-air laboratory test field on the test day. Rainy weather and muddy ground were intentionally selected as adverse test conditions (Figure 4).

To establish a baseline for comparison, a standalone encoder-based dead-reckoning trajectory (not intended for deployment) was translated so that its initial position coincided with the initial point of the surveyed reference path. The point-to-path deviation at time step *k* is defined as

(14) $$e_{k}=\min_{p\in P_{\mathrm{ref}}}{∥{\hat{p}}_{k}-p∥}_{2}$$

where ${\hat{p}}_{k}$ denotes the estimated position and $P_{\mathrm{ref}}$ represents the piecewise-linear surveyed reference path. Distances were evaluated against the line segments joining consecutive reference-path samples, avoiding dependence on their sampling density. Aggregate path deviation is quantified using the root mean square error (RMSE):

(15) $$\mathrm{RMSE}=\sqrt{\frac{1}{N}\sum_{k=1}^{N}e_{k}^{2}}$$

### 3.2. Odometry Behavior and Drift Characteristics

**Figure 5:**
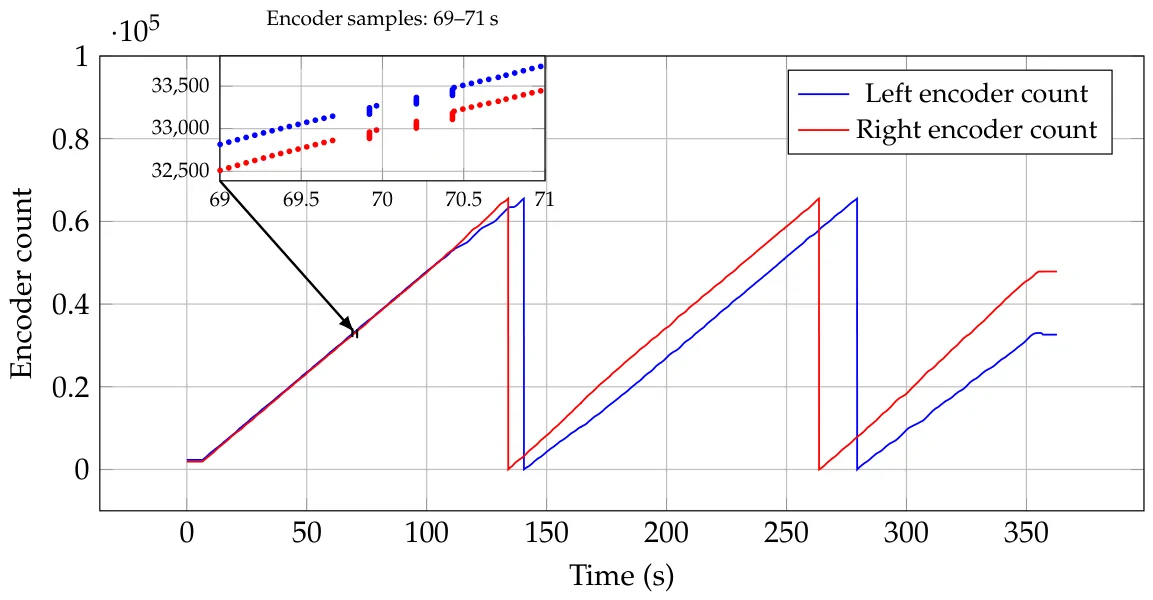
Raw encoder counts from the log files. Counter wraparounds occur near 134, 141, 264, and 279 s. The dashed region and arrow identify the source of the inset, which provides a sample-level view of both encoder signals between 69 and 71 s; individual measurements are shown as unconnected dots.

We first analyze encoder-only behavior to highlight the inherent limitations of dead reckoning. Figure 5 shows the raw encoder signals, including counter wraparounds near 134, 141, 264, and 279 s, which arise from unwrap handling of the raw counter values in the logging pipeline rather than from the underlying sensing hardware. The inset provides a sample-level view of both encoder signals during 69–71 s, with dots denoting the individual measurements. If left uncorrected in the odometry computation, such artifacts can contribute to drift accumulation in odometry-based localization.

The enlarged interval shows occasional encoder-update gaps of approximately 0.22–0.25 s, compared with the normal sampling interval of about 0.053 s, attributable to intermittent delays in the communication/logging pipeline rather than the encoder hardware itself. These delays temporarily provide stale track-speed information, which can introduce errors in the estimated traveled distance and heading, particularly during turning. Although the EKF limits these effects using IMU and GNSS measurements, short-term pose uncertainty may increase, especially if encoder delays coincide with degraded GNSS reception.

**Figure 6:**
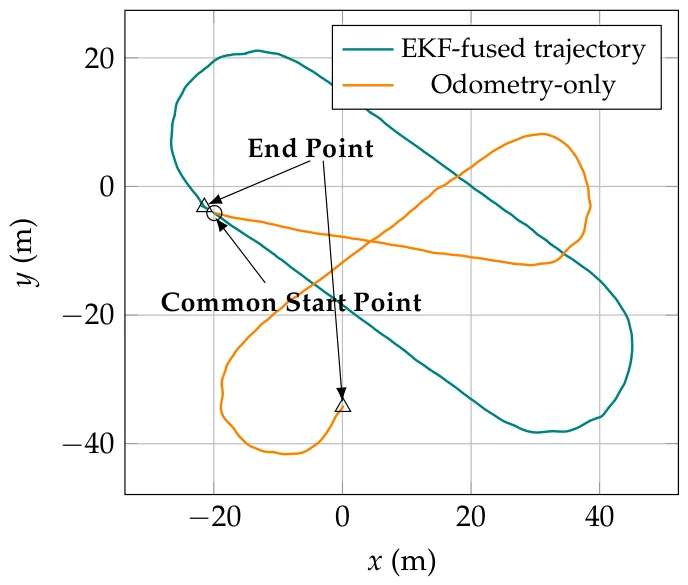
Encoder-only trajectory compared with the EKF-fused trajectory after initial-point alignment. A constant translation is applied to the odometry trajectory so that both trajectories share the same starting point; the subsequent divergence highlights cumulative drift in dead-reckoning localization.

After initial-point alignment, Figure 6 compares the encoder-only trajectory with the EKF-fused trajectory. A clear and systematic divergence is observed, particularly after consecutive turning maneuvers. This divergence illustrates accumulated encoder-only drift over the evaluated route.

### 3.3. IMU–Odometry Complementarity Analysis

**Figure 7:**
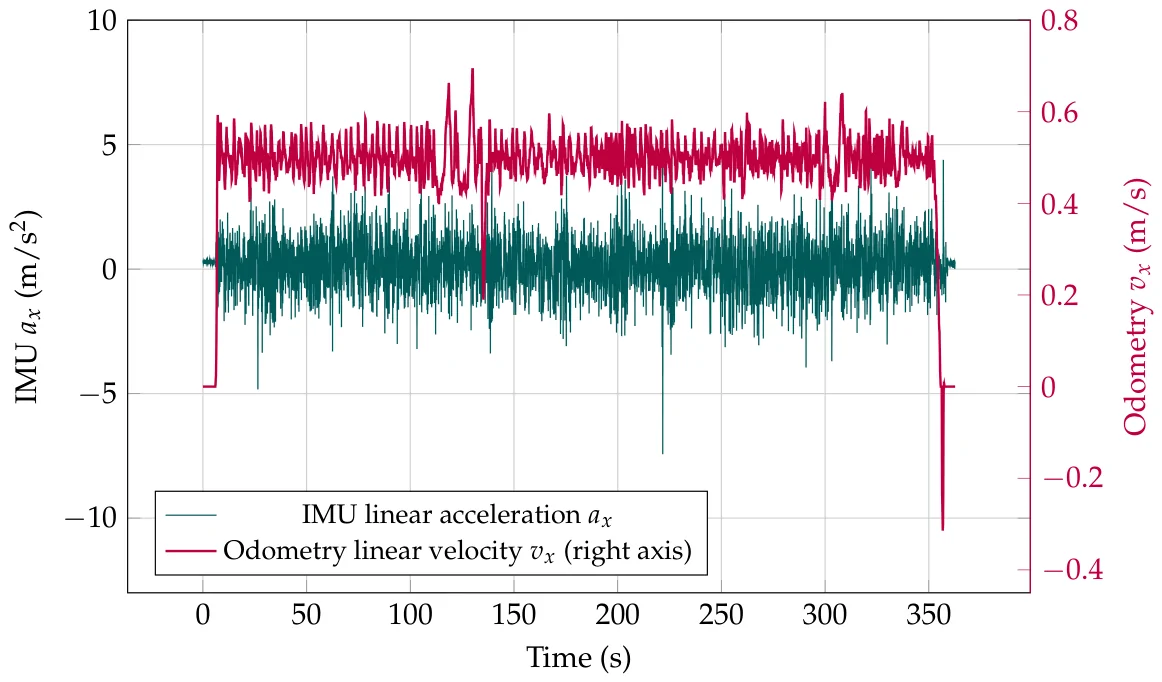
Comparison of IMU linear acceleration and odometry-derived longitudinal velocity. The signals show qualitative temporal correspondence during motion events; direct amplitude agreement is not expected because acceleration and velocity are different physical quantities.

**Figure 8:**
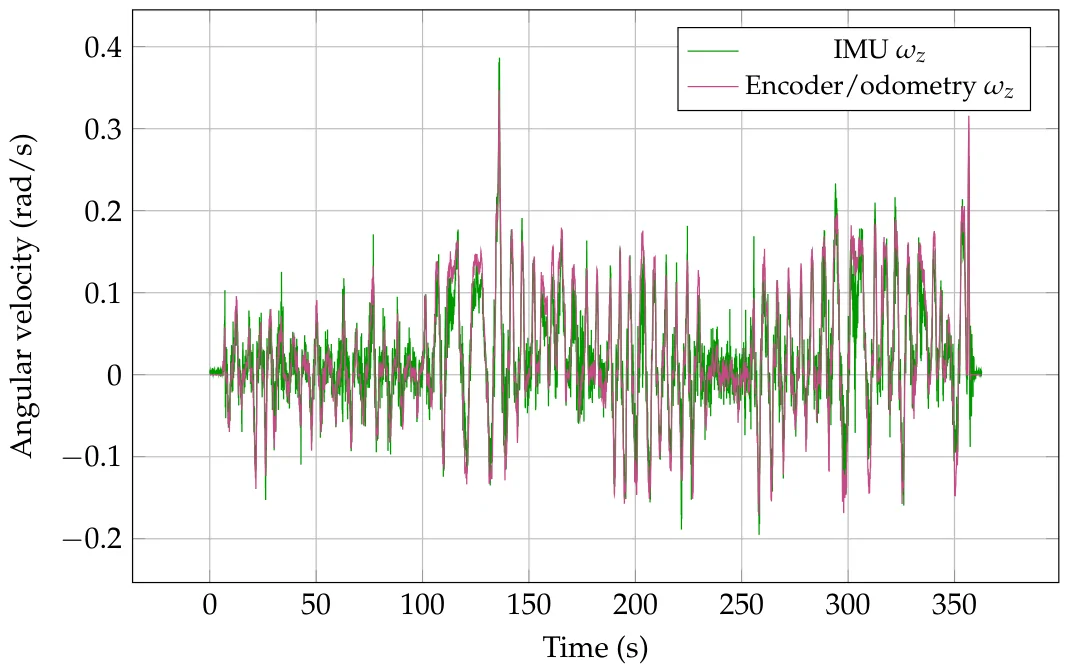
Angular velocity comparison between IMU and odometry signals. Agreement during turning maneuvers highlights complementary heading information.

Figure 7 and Figure 8 compare the IMU measurements with odometry-derived quantities. The signals show qualitative temporal correspondence, particularly during motion onset and turning events. Amplitude discrepancies and local transients are observed, but their individual causes are not established by these signal comparisons.

These observations confirm that the IMU and odometry measurements provide complementary information: odometry offers smooth velocity estimates under ideal conditions, while the IMU captures rapid dynamic changes. These comparisons motivate fusion but do not establish the standalone localization performance of the IMU.

### 3.4. GNSS-Only Performance and Limitations

**Figure 9:**
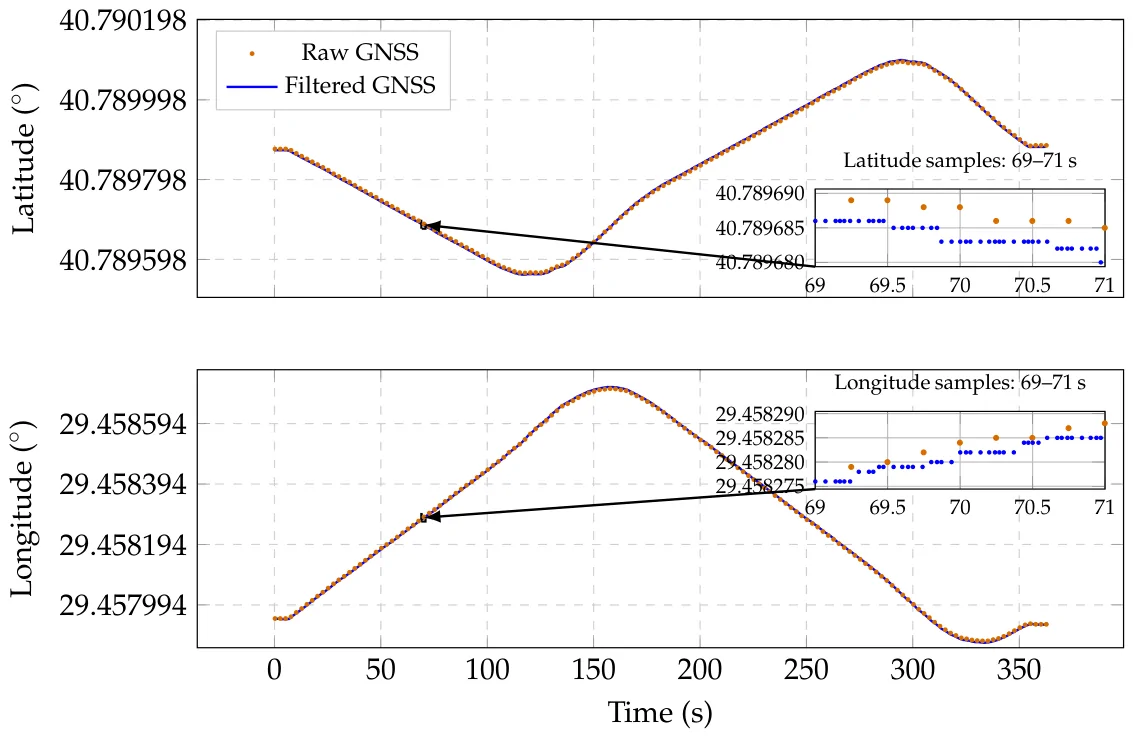
Raw and filtered GNSS latitude and longitude measurements. Raw GNSS samples are sparse and noisier, whereas the filtered GNSS trajectory is denser and smoother, highlighting the effect of GNSS preprocessing under degraded reception conditions. The dashed regions and arrows identify the 69–71 s latitude and longitude intervals enlarged in the corresponding insets, where individual full-resolution samples are shown as unconnected dots.

Figure 9 compares the recorded raw and filtered GNSS latitude and longitude measurements. The filtered signal follows the same overall coordinate evolution while providing a smoother and denser estimate. The insets enlarge the 69–71 s interval to show the sample-level differences between the two signals. Based on the full-resolution logs, the effective average update rates are approximately 4.0 Hz for the raw GNSS stream and 20.7 Hz for the filtered stream.

GNSS degradation motivates multi-sensor fusion in outdoor environments [7,8].

### 3.5. EKF Fusion Performance

**Figure 10:**
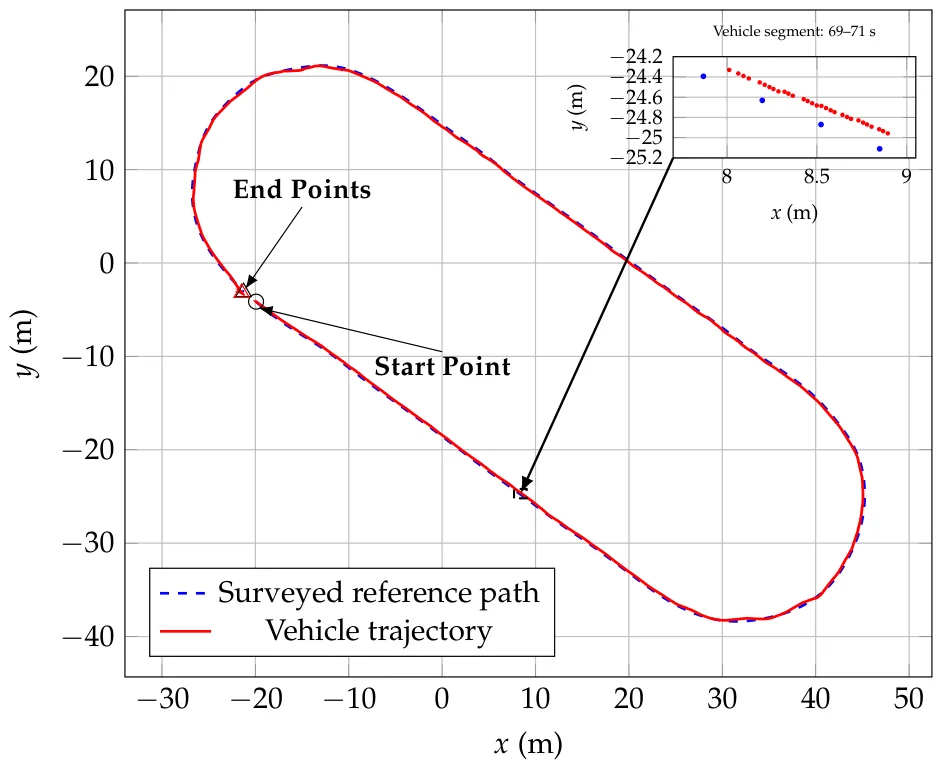
Vehicle trajectory from the filtered map-position estimate compared with the surveyed reference path. The close agreement throughout the route indicates small point-to-path deviation. The inset shows individual vehicle samples from 69–71 s together with nearby reference-path samples.

Figure 10 compares the original field-test EKF trajectory with the surveyed reference path, while the inset shows the recorded 69–71 s segment. The fused trajectory remains close to the reference path along both straight and high-curvature segments, whereas encoder-only odometry accumulates substantial drift.

Table 2 summarizes the quantitative results for the original recorded trajectory. Relative to the surveyed reference path, the EKF framework reduces the path-deviation RMSE from 12.248 m for initial-point-aligned odometry to 0.139 m, representing a reduction of approximately 98.9%. The P95 path deviation similarly decreases from 24.919 m to 0.213 m. These end-to-end metrics cover the complete original run, for which the effective raw-GNSS update rate was approximately 4.0 Hz. Thus, the complete fusion system produced substantially lower path deviation than initial-point-aligned encoder odometry in this run.

### 3.6. Robustness Analysis in Non-Ideal Conditions

A time-window analysis characterizes localization behavior within the evaluated run. For encoder-only odometry, the path-deviation RMSE increases from 6.181 m during 0–60 s to 21.324 m during the final 300–363 s interval, indicating substantial accumulated drift.

In contrast, the EKF-based solution maintains path-deviation RMSE values between 0.118 m and 0.155 m across the same intervals. This result characterizes the stability of the complete fusion system but does not isolate the contribution of covariance adaptation because a matched fixed-covariance EKF baseline was not evaluated. Future work will compare fixed and adaptive covariance settings using identical sensor inputs, initialization, models, and evaluation procedures.

### 3.7. Offline GNSS-Degradation Replay

**Figure 11:**
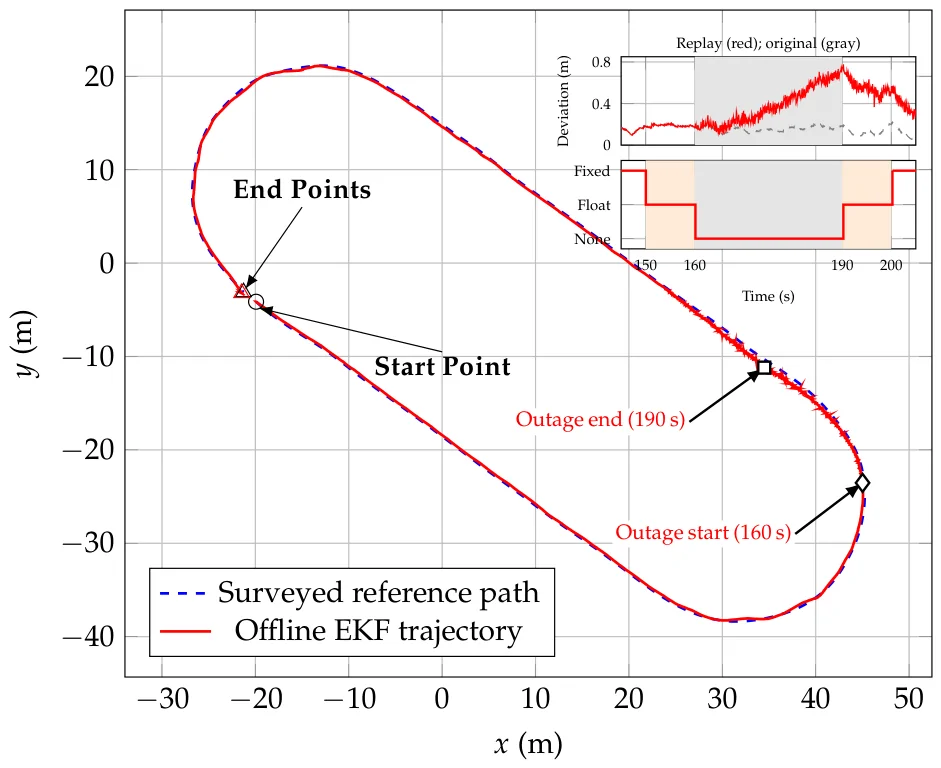
Trajectory obtained by rerunning the EKF offline with synthetically degraded GNSS inputs, compared with the surveyed reference path. The aligned insets show the point-to-path deviation for the replay in red and the original recording in gray, together with the imposed GNSS status. Shading marks the nominal degradation intervals from 150 to 160 and 190 to 200 s and the outage interval from 160 to 190 s in the status panel. The outage is also shaded in the deviation panel. The trajectory is an offline filter output. The GNSS degradation and outage are simulated rather than recorded field events. The diamond and square on the main trajectory identify the replay samples nearest the nominal outage start at 160 s and outage end at 190 s, respectively.

Figure 11 presents the trajectory obtained by rerunning the EKF offline with synthetically degraded GNSS inputs, including a simulated outage from 160 to 190 s. This evaluation concerns the injected scenario rather than an outage observed during field operation.

The main trajectory in Figure 11 marks the spatial locations of the replay samples nearest the nominal outage start at 160 s and outage end at 190 s, allowing the time-domain outage interval to be related directly to the corresponding segment of the estimated path.

Table 3 quantifies how path deviation changes across the imposed RTK-status phases shown in Figure 11. The fixed, float, and no-RTK intervals represent nominal operation, degraded reception or reacquisition, and the synthetic outage, respectively. Comparing RMSE, P95, and maximum deviation before, during, and after the outage shows both error growth and recovery behavior. The intervals follow the imposed status-transition times and are not field-observed RTK events.

Unlike aggregate metrics, the time-domain view in Figure 11 reveals when deviation increases and how it persists after the simulated outage, without attributing individual peaks to unverified encoder, vibration, or terrain events.

To quantify sensitivity to outage duration, controlled offline replays were additionally performed with synthetic GNSS-denial intervals of 30, 40, 50, and 60 s. In every replay, the same recorded sensor sequence, EKF initialization, motion and measurement models, covariance settings, and surveyed reference path were used. The weakening interval before denial and the 10 s reacquisition interval were also held fixed; only the GNSS-denial duration was changed. These tests are simulated robustness evaluations, not additional field experiments.

Table 4 shows monotonic error growth over the tested durations. As the outage increased from 30 to 60 s, degraded-window RMSE increased from 0.421 to 0.773 m, degraded-window P95 increased from 0.668 to 1.303 m, and peak deviation increased from 0.766 to 1.417 m. Whole-run RMSE also increased from 0.208 to 0.409 m. The results support distinguishing short and prolonged outage regimes, but they do not establish that 30 s is the optimal switching threshold or demonstrate equivalent performance during real field outages.

## 4. Discussion

The field results show that the complete EKF fusion system produced lower path deviation than encoder-only dead reckoning during the evaluated run. The separate synthetic GNSS-degradation replay illustrates the temporal growth of deviation and the subsequent recovery under injected disturbances. It does not demonstrate rejection of all disturbance mechanisms that may occur in the field. Encoder-only dead reckoning therefore provides a baseline for the complete fusion system rather than an isolated test of covariance adaptation. The lower path-deviation RMSE and P95 support the benefit of fusion within this run.

The tracked undercarriage presents motion-estimation challenges that differ from ideal rolling-wheel motion. In Equation (5), the mean track displacement determines translation and the difference between the track displacements determines yaw. Unequal track velocities produce turning, while unequal slip causes the encoder-derived increments to differ from the actual body motion. Skid steering and intermittent ground contact can also generate lateral displacement that is not represented by the planar model. Terrain disturbances and vibration affect the cabin-mounted IMU, while the roof-mounted GNSS antenna measures a position offset from the undercarriage reference.

The EKF uses encoder increments for motion prediction, IMU yaw measurements to constrain heading, and GNSS position measurements to limit accumulated drift. Encoder preprocessing suppresses fluctuations and rejects extreme speed values, but it cannot identify all physically plausible slip. Process-covariance inflation and the down-weighting of degraded GNSS measurements change the relative influence of prediction and measurement updates. They do not directly estimate slip, vibration, or track–terrain interaction. The measured swing angle and calibrated sensor offsets are applied through the URDF/TF chain to transform the upper-structure measurements continuously into the undercarriage base_link frame. Equation (6) includes these time-varying transformation terms.

These observations are consistent with earlier studies showing that RTK-GNSS performance degrades under partial obstruction [7,8] and that multi-sensor integration improves localization continuity in outdoor robotic systems [3,27]. Table 5 places the present work alongside selected construction-machine localization, perception, pose-estimation, and navigation studies. The numerical results cannot be ranked directly because machine localization error, estimator consistency, articulated-keypoint error, traversability-map accuracy, and navigation success rate describe different quantities and use different reference protocols.

Relative to existing GNSS/IMU/odometry systems [25,27], the contribution of this work is the integration and field evaluation of a minimal undercarriage estimator on a tracked excavator. The system uses GNSS-RTK, IMU, and track encoders without a LiDAR localization map or external localization infrastructure. The surveyed path is used only as an evaluation reference and is not supplied to the EKF.

Several limitations constrain the interpretation of the results. The field validation consists of an approximately 363 s run on a predefined route. The straight and turning segments and the reported time windows describe variation within that run rather than independent trials. The synthetic GNSS replay is also not an additional field experiment. Consequently, the observed trajectory continuity does not establish repeatability across excavation cycles or reliability under untested disturbances. Broader validation requires repeated trials across machines, routes, terrain and weather conditions, GNSS availability, and operational motion patterns.

The framework also relies on manually tuned covariance parameters and does not explicitly model slip dynamics. The minimal three-state EKF estimates only *x*, *y*, and θ, which supports computational efficiency and real-time operation on embedded hardware but excludes explicit velocity, IMU bias, and slip states. The contribution therefore lies in system integration and field evaluation on the tested excavator rather than in an algorithmic advance. Extending the state vector and introducing slip-aware motion models may improve performance under more severe terrain conditions.

The evaluation reference imposes a further limitation. Although the reference path was established independently through surveying and mapping, it is a static geometric path rather than a time-synchronized ground-truth trajectory. The point-to-path metrics therefore do not capture along-path timing errors and do not measure time-synchronized absolute localization error. The coordinate-conversion discrepancy is also non-negligible relative to the 0.139 m path-deviation RMSE, although it is not a direct correction to that metric. Its effect depends on whether the estimated trajectory and the surveyed reference path use the same transformation. Centimeter-level geodetic consistency requires an ellipsoidal ENU or suitable projected-coordinate transformation and consistent registration of the trajectories and reference path, including datum and height conventions.

Urban-canyon operation was not evaluated experimentally. Tall buildings can reduce satellite visibility and introduce multiple paths, which may degrade the RTK position and heading or prolong outages. The filter down-weights degraded GNSS measurements and continues encoder prediction and IMU heading updates during outages. These mechanisms do not guarantee bounded drift or rejection of multipath biases. The synthetic scenario in Figure 11 does not replace field validation near tall buildings.

Future work should address these limitations through repeated field trials, controlled comparisons of fixed and adaptive covariance settings, explicit slip-aware motion modeling, and integration of additional sensing modalities. Machine learning-enhanced sensor fusion may also help adapt the estimator to time-varying environmental conditions and sensor reliability [32]. Hybrid EKF–learning frameworks could combine model-based estimation with data-driven adaptation in highly dynamic construction environments.

**Table 5:** Comparison of the selected localization, mapping, pose-estimation, perception, and navigation studies. Reported numerical results use different target quantities and reference protocols and therefore do not constitute a direct accuracy ranking.

Study	Estimated Quantity and Method	Reported Numerical Result	Experimental Platform	Comparability and Scope
Nubert et al. [3]	Six-degrees-of-freedom machine pose; GNSS, IMU, and LiDAR fused in dual-factor graphs	Translational estimator consistency: 0.58 cm mean and 3.16 cm standard deviation	Two walking excavators in representative operational environments	Directly relevant localization study, but consistency between estimator outputs is not independent absolute-position error.
Guan et al. [33]	Terrain traversability and navigation; RGB imagery, Livox LiDAR point clouds, semantic–geometric mapping, planning, and control	Trajectory-tracking error ≤10 cm (average error 0.059–0.104 m across straight and turning paths); mapping accuracy improved by 4.17–30.48%	49-ton autonomous excavator on outdoor sites containing pits, slopes, rocks, and uneven terrain	Provides quantitative perception/navigation performance, but no chassis-localization error comparable with RMSE or P95.
Di et al. [34]	Dump truck 3D pose and bounding box relative to excavator base; point-cloud feature matching (PPF global descriptors) with Hesai QT-64 LiDAR and EVS camera	Reported positioning accuracy of ±10 cm; no RMSE, percentile, or common reference protocol was reported	Full-scale unmanned-excavator field experiments	Focuses on target/vehicle perception (dump truck pose relative to excavator) during loading, rather than global chassis trajectory estimation.
Schmidt and Berns [35]	Pose-to-pose construction-site navigation; 2D grid mapping, extended A*, obstacle enlargement, and forward/reverse path following	No aggregate localization-error statistic reported; validation demonstrated planned-path execution and repositioning	18-ton Volvo EW180B wheeled excavator, THOR, in construction-site experiments	Navigation study that assumes an available machine pose; it does not evaluate a localization estimator against ground truth.
Tang et al. [36]	Three-dimensional articulated keypoints; onboard cameras, IMUs, kinematic constraints, and EKF-based competitive/complementary fusion	3D keypoint location RMSE of 73.76 mm (independent motion) and 90.66 mm (continuous motion) on medium excavator; boom K3 RMSE ∼54.9–61.3 mm, arm K4 RMSE ∼78.0–179.9 mm on large excavator	Hitachi ZAXIS 240 excavator in real-machine motion tests	Quantifies articulated full-body pose relative to the sensing frame, not global undercarriage trajectory error.
Jud et al. [28]	Full 3D robot state (chassis/cabin pose, joint states, velocity); EKF integrating GNSS-RTK, cabin IMU, joint sensors, wheel rolling prediction, and legged kinematics	Demonstrated autonomous tasks: embankment excavation within 0.03 m average error, stone placement error of 0.128 m (gripper placement error <30 cm)	HEAP (Menzi Muck M545) autonomous walking excavator with active chassis balancing and hydraulic legs	Demonstrates localization embedded in a complete walking-excavator autonomy stack (trenching, stone walling, forestry); does not report a standalone chassis path-deviation statistic.
This work	Planar undercarriage pose (x,y,θ); extended Kalman filter (EKF) fusing GNSS-RTK, IMU, and left/right track encoders with swing-angle-based TF frame transformations	Path-deviation RMSE of 0.139 m and P95 of 0.213 m relative to an independently surveyed static reference path (98.9% RMSE reduction vs. raw odometry)	14-ton HKM 140LC tracked hydraulic excavator tested in rainy weather on muddy outdoor terrain	Evaluates an onboard minimal undercarriage tracking system on a ∼363 s route without LiDAR maps; metric is geometric point-to-path spatial deviation rather than time-synchronized absolute pose error.

## 5. Conclusions

This study developed and experimentally evaluated an EKF-based localization framework for a tracked autonomous excavator. The framework integrates GNSS-RTK, IMU, and track-encoder measurements within an ROS pipeline. Its principal engineering contribution is the integration of these measurements with swing-angle-based coordinate transformations that express upper-structure sensor data in the undercarriage frame during operation.

During the approximately 363 s outdoor field experiment, the fused trajectory achieved a path-deviation RMSE of 0.139 m and a P95 path deviation of 0.213 m relative to the independently surveyed reference path. Encoder-only odometry produced an RMSE of 12.248 m and a P95 deviation of 24.919 m. The complete fusion system therefore reduced path-deviation RMSE by approximately 98.9% and maintained a trajectory close to the reference path across both straight and turning segments.

The GNSS-outage robustness analysis was based on controlled offline synthetic replays rather than naturally occurring field outages. The results showed increasing deviation as outage duration increased, followed by recovery behavior after GNSS measurements became available again. Together, the field and replay results demonstrate the practical value of combining absolute, inertial, and proprioceptive measurements for continuous excavator undercarriage localization in non-ideal outdoor conditions.

The proposed framework provides a computationally compact localization solution that can be implemented using onboard sensors without a LiDAR localization map or external positioning infrastructure. Future development will focus on broader field deployment, slip-aware state estimation, systematic covariance analysis, and the integration of additional sensing modalities to support increasingly demanding autonomous excavation tasks.

## Figures and Tables

**Table 1:** The hardware content of the test platform.

No.	Name
1	MTi-680G RTK GNSS/INS module
2	3-axis gyroscope (MTi-680G)
3	3-axis accelerometer (MTi-680G)
4	3-axis magnetometer (MTi-680G)
5	GNSS antenna (RTK-enabled)
6	Wavlink wireless router
7	Quadrature encoders (Pallets)
8	Industrial computer (ROS)

**Table 2:** Point-to-path deviation relative to the surveyed reference path.

Method	Mean (m)	RMSE (m)	P95 (m)	Max (m)
Odometry-only (initial-point-aligned)	10.094	12.248	24.919	26.069
EKF-fused	0.130	0.139	0.213	0.280

**Table 3:** Path-deviation statistics for each imposed RTK-status phase of the offline replay, comparing localization error before, during, and after the synthetic GNSS outage. Intervals include their start and exclude their end, except for the final sample.

Status	Interval (s)	Samples	RMSE (m)	P95 (m)	Max (m)
Fixed	0.0000–150.0340	2251	0.130	0.198	0.280
Float (degraded)	150.0340–160.1316	151	0.188	0.205	0.211
No RTK (outage)	160.1316–190.2494	452	0.418	0.685	0.750
Float (degraded)	190.2494–200.2495	150	0.561	0.694	0.778
Fixed	200.2495–362.6636	2437	0.163	0.273	0.570

**Table 4:** Path deviation for controlled offline replays with different synthetic GNSS-outage durations. All error values are in meters. Window metrics cover the degraded interval from outage onset through the imposed drift-recovery period.

Outage (s)	Full-Run RMSE	Full-Run P95	Window RMSE	Window P95	Window Peak
30	0.208	0.491	0.421	0.668	0.766
40	0.261	0.658	0.530	0.861	1.021
50	0.328	0.874	0.648	1.069	1.157
60	0.409	1.070	0.773	1.303	1.417

## Data Availability

The data presented in this study are available on request from the corresponding author.
